# ELTD1 promotes invasion and metastasis by activating MMP2 in colorectal cancer

**DOI:** 10.7150/ijbs.62293

**Published:** 2021-07-13

**Authors:** Jiawei Sun, Zizhen Zhang, Jingyu Chen, Meng Xue, Xia Pan

**Affiliations:** 1 Department of Gastroenterology, the Second Affiliated Hospital of Zhejiang University School of Medicine, Hangzhou 310020, China; 2 Shulan International Medical College, Zhejiang Shuren University, Hangzhou 310015, China.; 3 Institution of Gastroenterology, Zhejiang University, Hangzhou 310000, China

**Keywords:** ELTD1, MMP2, invasion, metastasis, colorectal cancer

## Abstract

Metastasis is a key factor that affects the prognosis of colorectal cancer (CRC), and patients with metastasis have limited treatment options and poor prognoses. EGF, latrophilin, and seven transmembrane domains containing 1 (ELTD1/ADGRL4) are members of the adhesion G protein-coupled receptor (aGPCR) superfamily. In this study, high expression of ELTD1 was correlated with lymph node metastasis and poor outcomes in CRC patients. Both in vitro and in vivo studies showed that ELTD1 markedly promoted the invasion and metastasis of CRC. Moreover, ELTD1 accelerated the transcriptional activity of MMP2, which could rescue the impaired invasiveness of CRC cells caused by the downregulation of ELTD1 expression. In conclusion, our study suggests that ELTD1 might be a potential novel target for the treatment of CRC metastasis.

## Introduction

Colorectal cancer (CRC) is the fourth most commonly diagnosed cancer and one of the leading causes of cancer-related death worldwide 1. According to recent studies, ulcerative colitis (UC) significantly increases the risk of CRC 2. Previously, we summarized the genes whose expression is altered during the formation of colitis-associated cancer (CAC); these genes are related to DNA repair, immune responses, cell metabolism and interactions with microbiota 3. Among these genes, epidermal growth factor, latrophilin and seven transmembrane domains containing 1 (ELTD1/ADGRL4), a markedly overexpressed gene in CAC, attracted our attention.

Initially discovered in 2001 4, ELTD1 is an orphan member of the adhesion G protein-coupled receptor (aGPCR) superfamily. The expression of ELTD1 is induced by VEGFR and TGF-β4,5 and repressed by DLL 5, 6. Although it is similar to the majority of adhesion GPCRs, the activation and signaling mechanisms of ELTD1 remain unknown 7. As a regulator of angiogenesis, ELTD1 could act as a potential therapeutic target for treating malignant tumors. The role of ELTD1 in angiogenesis has already been verified in breast cancer, clear cell renal cell carcinoma, head and neck squamous cell carcinoma, retinoblastoma and glioma 8-12. Pekow J et al 13 found that ELTD1 expression was significantly upregulated in UC patients with neoplasia compared to those without neoplasia and healthy controls. Masiero M et al 10 reported that ELTD1-silenced CRC cells grew slowly in xenograft tumors, while CRC patients with higher ELTD1 expression in tumor-associated endothelial cells had better prognosis. To interpret this contradiction, it is necessary to comprehensively explore the effect of ELTD1 on the carcinogenesis and progression of CRC.

In this study, we investigated the impact of ELTD1 on the progression of CRC, focusing on CRC invasion and metastasis. In addition, downstream targets of ELTD1 were screened, and the regulatory molecular mechanisms were explored. These findings may help us to develop new biological agents that target ELTD1 in CRC in the future.

## Methods

### Establishment of the CAC mouse model

Six-week-old male C57BL/6J mice (n=10) were obtained from Shanghai Silaike Animal Biotechnology Co, Ltd. Half of these mice were used to establish a CAC model by the intraperitoneal injection of azoxymethane (AOM) (10.0 mg/kg body weight; Sigma-Aldrich, USA). Then, the mice received 3 cycles of 2.5% (wt/vol) dextran sulfate sodium (DSS) (MP Biomedicals, USA) in their drinking water for 7 days followed by normal drinking water for 16 days. The other half of the mice were used as negative controls and intraperitoneally injected with normal saline and provided drinking water alone 14.

### Cell culture

CRC cells (HT29, RKO and HCT116 cells) were cultured in McCoy's 5A medium or EMEM supplemented with 10% fetal bovine serum. The cells were maintained in a 37°C incubator with 5% CO_2_ humidified air.

### Cell transfection

The MMP2-expression plasmid (Sino Biological, Beijing, China) or ELTD1 siRNAs (Shanghai GeneChem, Shanghai, China) were transfected into CRC cells with Lipofectamine 2000 (Invitrogen, Carlsbad, CA) for the induction or silencing of the indicated genes, and the transfection efficiency was analyzed by qRT-PCR and Western blotting 72 h later. ELTD1 siRNA sequences were as follows: sense, GCUUCAGAUCCAGCAGUAATT; antisense, UUACUGCUG GAUCUGAAGCTT.

### Transwell assay

Transwell assays were performed to measure the migratory and invasive abilities of cells in vitro. In the migration assay, a total of 3×10^5^ CRC cells were plated in the top chamber of the Transwell plate (24-well insert, pore size: 8 mm, Corning, Life Sciences, USA) with serum-free medium, while the medium in the lower chamber contained 10% FBS. After incubation for 18 h, the migrated cells were fixed, stained with DAPI, and then counted in 5 random fields by fluorescence microscopy. In the invasion assay, 3×10^5^ CRC cells were plated in the top chamber coated with 20 μg Matrigel (BD Biosciences, San Jose, CA, USA). After 24 h, the invaded cells were fixed, stained in 0.05% crystal violet solution, and then observed under an inverted microscope. In addition, the absorbance of the cell eluent was detected at 562 nm.

### Western blotting

Proteins were extracted from HT29, RKO and HCT116 cells and separated on Tris-glycine gels, and Western blotting analyses were performed according to standard procedures. The protein extracts were immunoblotted with an ELTD1 antibody (1:1000, Genetex, USA) and an MMP2 antibody (1:1000, Abcam, USA). β-actin (antibody at 1:1000, Genetex, USA) was used as an internal control.

### Quantitative reverse-transcription PCR

Total RNA was isolated from colon tissues or CRC cells using TRIzol reagent (TaKaRa Bio, Japan). ELTD1 expression was then measured in triplicate using SYBR Green qPCRMix (TaKaRa Bio, Japan). Primer sequences were as follows: ELTD1 Forward: 5´-CTCAGTCCTGTGGCGAAAATG3´, ELTD1 Reverse: 5´-GGTTACTGCTGGATCTGAAGC-3´; MMP2 Forward: 5´-TGACTTTCTTGGATCGGGTCG-3´, MMP2 Reverse: 5´-AAGCACCACATCAGATGACT-3´; GAPDH Forward: 5´-CTCACCGGATGCACCAATGTT-3´, GAPDH Reverse: 5´-CGCGTTGCTCACAATGTTCAT-3´.The 2^-ΔCT^ method was used to calculate relative mRNA expression.

### Clinical samples

CRC patients with detailed clinical and pathological data who underwent surgery between July 2014 and December 2019 at the Second Affiliated Hospital, School of Medicine, Zhejiang University were enrolled. Tumor tissues and adjacent nontumorous tissues from 86 CRC patients were fixed, embedded in paraffin wax, and then sectioned for further use.

### Immunohistochemical analysis

Tissue sections were incubated at 60°C for 30 minutes, followed by conventional dewaxing and antigen repair. Tissue slides were sequentially incubated with polyclonal rabbit anti-ELTD1 antibodies (Genetex, USA) at a 1:1000 dilution at 4°C overnight. Then, 3,3-diaminobenzidine (DAB) was added and incubated in the dark at room temperature for 1 min. Protein expression was observed under a microscope by two independent pathologists. The histologic score (H-score) was calculated with the following formula: H-Score=(percentage of cells with weak intensity×1)+(percentage of cells with moderate intensity×2)+percentage of cells with strong intensity ×3). Based on the H-score result, ELTD1 expression was classified as low (H-score < 100) or high (H-score ≥ 100) 15.

### Luciferase activity assay

A fragment ranging from -1902 bp upstream to +98 bp downstream of the MMP2 transcription start site was obtained and inserted into the reporter gene vector GV238 to create the MMP2 luciferase plasmid (Shanghai GeneChem, Shanghai, China). ELTD1 siRNA was cotransfected with the MMP2 luciferase reporter plasmid into HT29, RKO and HCT116 cells. The Dual-Luciferase Reporter Assay System (Promega) was used to measure the luciferase activity after 48 h.

### Establishment of the lung metastasis model

HCT116 cells were transfected with CMV-GFP lentivirus encoding ELTD1 and the puromycin selection box to obtain cell lines stably overexpressing ELTD1. To establish a lung metastasis model, 1.5×10^6^ HCT116 cells (negative control or ELTD1-overexpressing cells) diluted in 200 μl PBS were injected into the caudal veins of 4- to 5-week-old female nude mice (n=10). The mice were weighed weekly and sacrificed 10 weeks after injection. Lung tissues were harvested and imaged using an in vivo imaging system (Thermo Scientific, USA) to detect fluorescence. Additionally, the weight of the metastatic nodules was compared between the two groups.

### Analysis of TCGA data

RNA-seq expression data and clinical information of CRC patients were downloaded from the cBioProtal (http://www.cbioportal.org/study?Id=- download TCGA coad tcea # summary) database. Then, survival curves of these patients (n=279) were generated using Kaplan‐Meier plots, and the correlation between the mRNA expression level of ELTD1 in tumor samples (n=77) and the N (local lymph node involvement) stage was analyzed.

### Statistical analysis

The data are expressed as the mean ± standard deviation (SD) from at least 3 independent experiments, and statistical analyses were performed with GraphPad Prism 6 and SPSS 20.0 statistical software. The chi-square test was used to assess the association of ELTD1 expression with clinicopathological parameters. Kaplan-Meier plots were compared with the log-rank test. Differences were considered statistically significant at a p value < 0.05.

## Results

### ELTD1 mRNA levels were upregulated in mice with CAC

**Figure 1:**
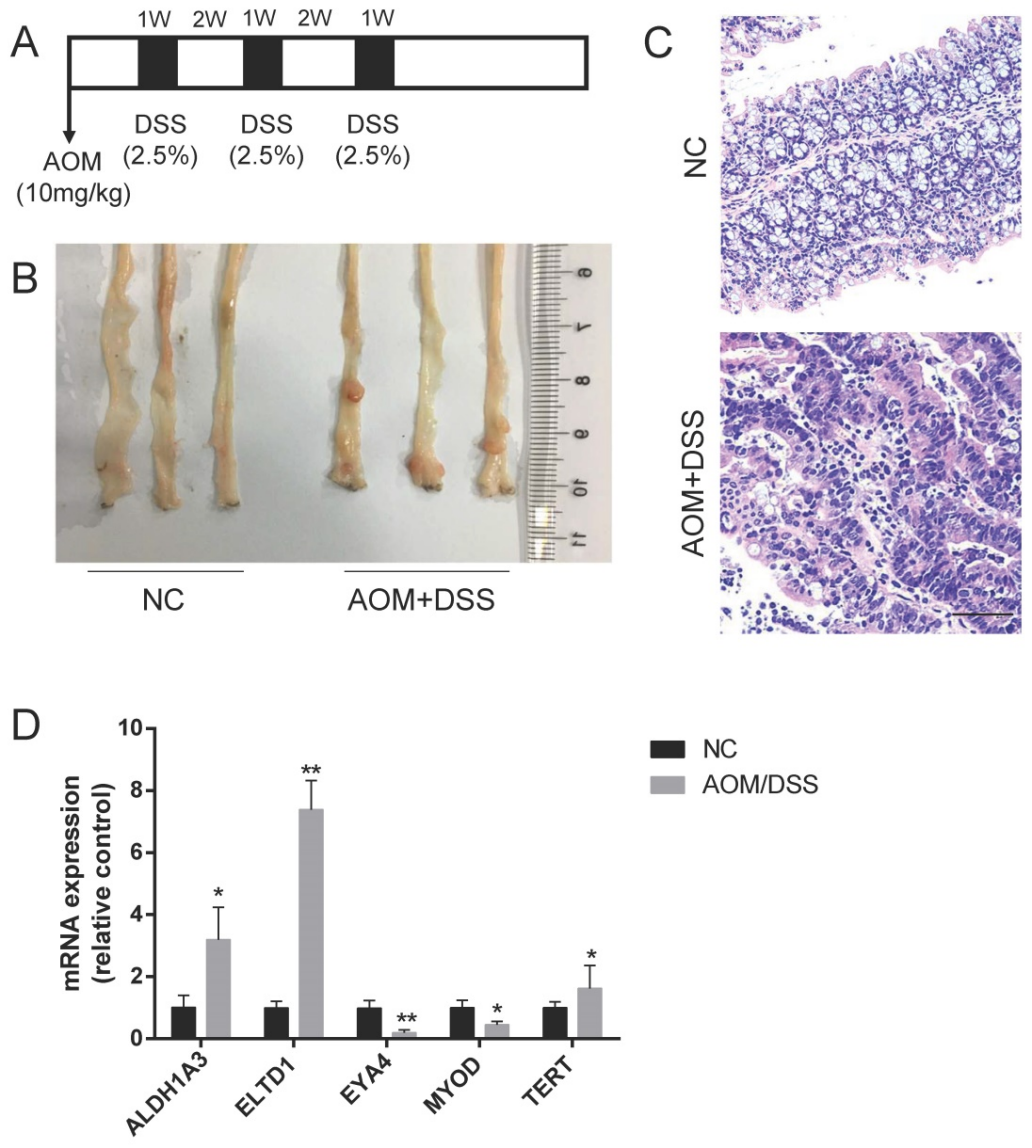
Upregulation of ELTD1 expression in colon tumors from AOM/DSS-treated mice. **(A)** Establishment of the AOM/DSS-induced CAC model. **(B, C)** Representative photographs of macroscopic and histological observations (H&E) in colon tissue. **(D)** qRT-PCR measurement of ALDH1A3, ELTD1, EYA4, MYOD, and TERT expression in tumor tissues from AOM/DSS-treated mice or colon tissues from negative control mice. The results are shown as the mean ± SD (n = 10 for each group), **p<0.01 and *p<0.05. Scale bar=100 μm.

Based on our previous review^3^, ALDH1A3, ELTD1, EYA4, MYOD and TERT expression was upregulated in CAC, but the roles of these molecules in CRC were not fully defined. To validate their expression in CAC, a CAC mouse model was established by the intraperitoneal injection of the mutagen AOM, followed by 3 cycles of DSS administration (Figure 1A). To ensure tumor formation, the mice were sacrificed 15 weeks after AOM injection, and 60% of the mice had developed well-differentiated adenocarcinomas in the distal colon (Figure 1B, C). We detected the mRNA expression of these selected genes and found that the expression of ELTD1 was most highly increased in the AOM+DSS mice (Figure 1D, p<0.01).

### ELTD1 was overexpressed and correlated with tumor progression in CRC

**Figure 2:**
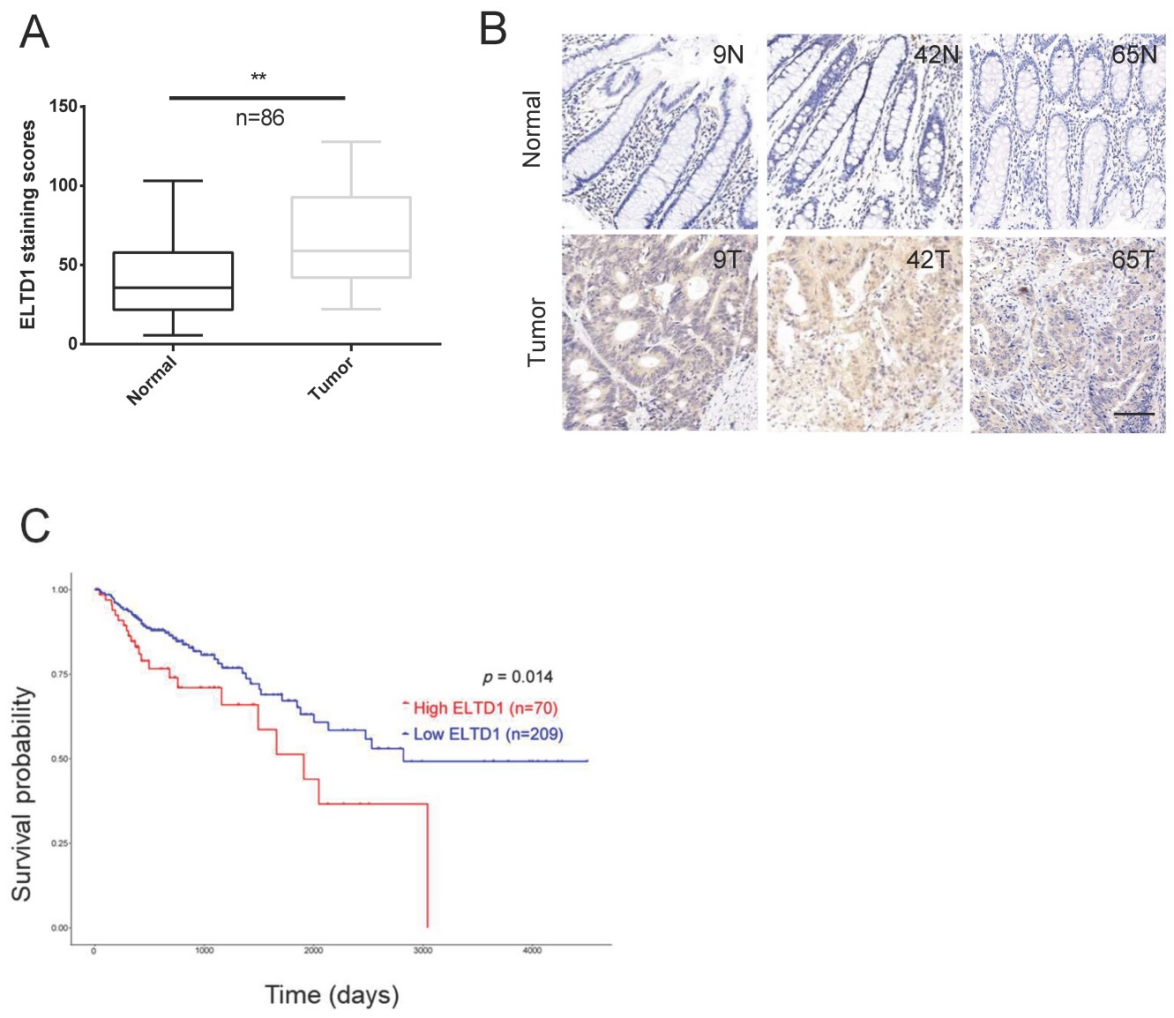
ELTD1 was highly expressed in CRC and predicted poor prognosis. **(A)** Relative ELTD1 expression levels in CRC tissues and adjacent normal CRC tissues as detected by qRT-PCR (n=86).** (B)** Representative images of IHC staining of ELTD1 in CRC tissues and adjacent normal tissues. **(C)** Survival probability curves based on the expression of ELTD1 in CRC patients (n=279). *p<0.05. Scale bar=100 μm.

Quantitative analysis indicated that the expression of ELTD1 was upregulated in the clinical CRC tissues compared with the corresponding adjacent nontumorous tissues (Figure 2A, B). The tumor node metastasis (TNM) system is the most extended scheme of stage grouping in cancer 16. ELTD1 expression was increased in the advanced T, N or M stages, and the difference between lymph node metastasis (N1+N2) and node-negative (N0) patients was significant (p<0.05) (Table 1, Supplementary Figure 1). Based on the median expression level of ELTD1, CRC patients (n=279) were divided into the high-ELTD1 group (n=70) and the low-ELTD1 group (n=209). The overall survival rate was worse for CRC patients with high ELTD1 expression (p=0.014) than for those with low ELTD1 expression (Figure 2C).

### Knockdown of ELTD1 suppressed the migration and invasion of CRC cells

**Figure 3:**
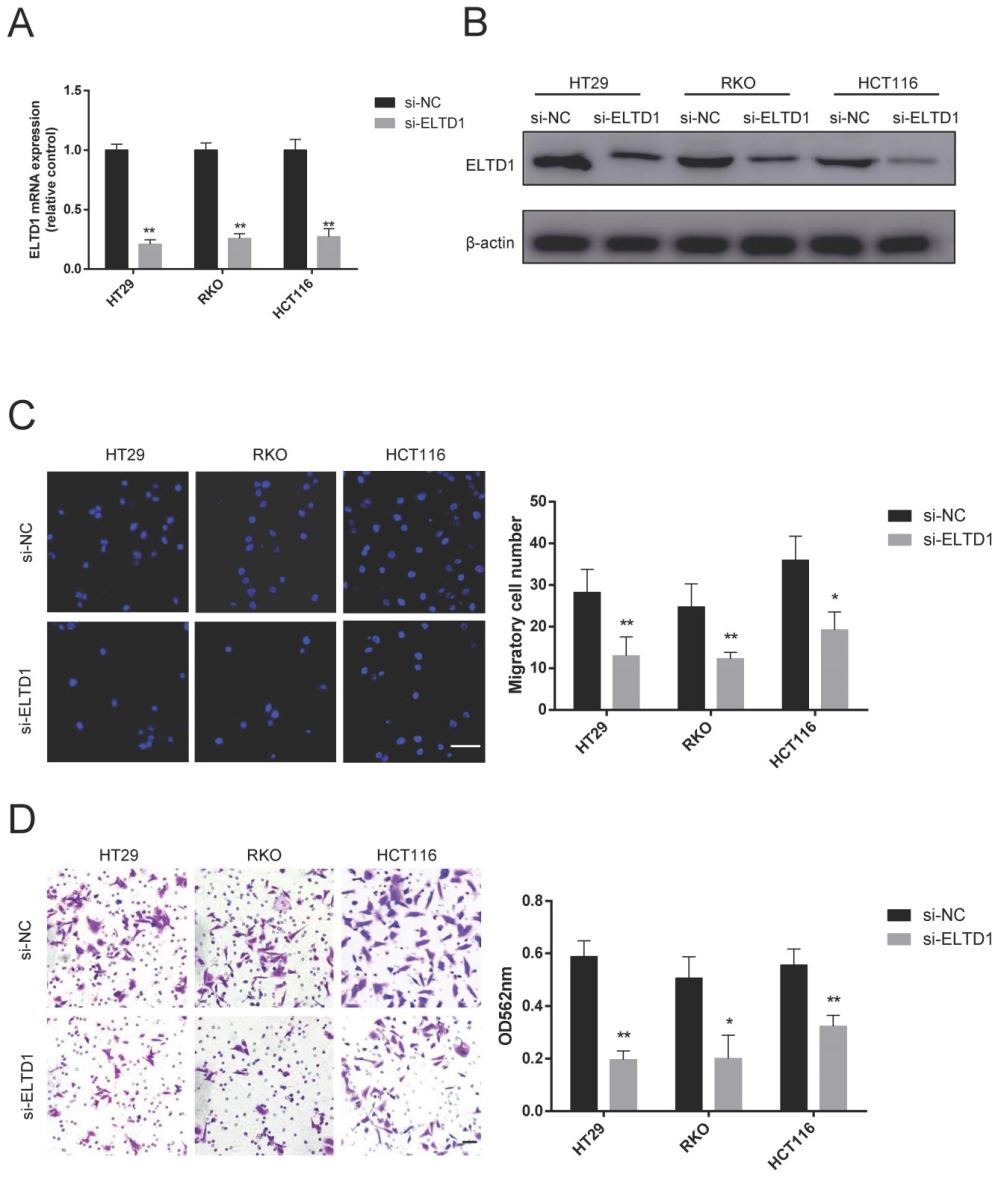
Knockdown of ELTD1 inhibited CRC cell migration and invasion. **(A, B)** The efficiency of ELTD1 siRNA in HT29, RKO and HCT116 cells was verified by qRT-PCR **(A)** and Western blotting **(B)**. **(C)** Representative DAPI staining and statistical results of the migration of ELTD1-silenced CRC cells in the Transwell assay. **(D)** Invasion abilities of the two CRC cell lines were assessed by Transwell assay, followed by crystal violet staining and statistical analysis. The results are shown as the mean ± standard deviation (SD) of three independent experiments. **p <0.01 and *p<0.05. Scale bar=100 μm.

First, the expression of ELTD1 in 6 human CRC cell lines was detected by qRT-PCR and Western blotting. HT29, RKO and HCT116 cells, which had a median level of ELTD1 expression, were chosen for further siRNA experiments to study both gain and loss of ELTD1 function (Supplementary Figure 1). Given the significant difference in N stage, rather than T stage, we focused on the malignant behavior of invasiveness. ELTD1 siRNA was transfected into HT29, RKO and HCT116 cells (Figure 3A, B). As shown by the Transwell assay, ELTD1 knockdown decreased the number of cells that migrated into the lower chamber (Figure 3C). In addition, fewer CRC cells invaded through the Matrigel after ELTD1 expression was inhibited (Figure 3D).

### Overexpression of ELTD1 promoted CRC cell migration and invasion

**Figure 4:**
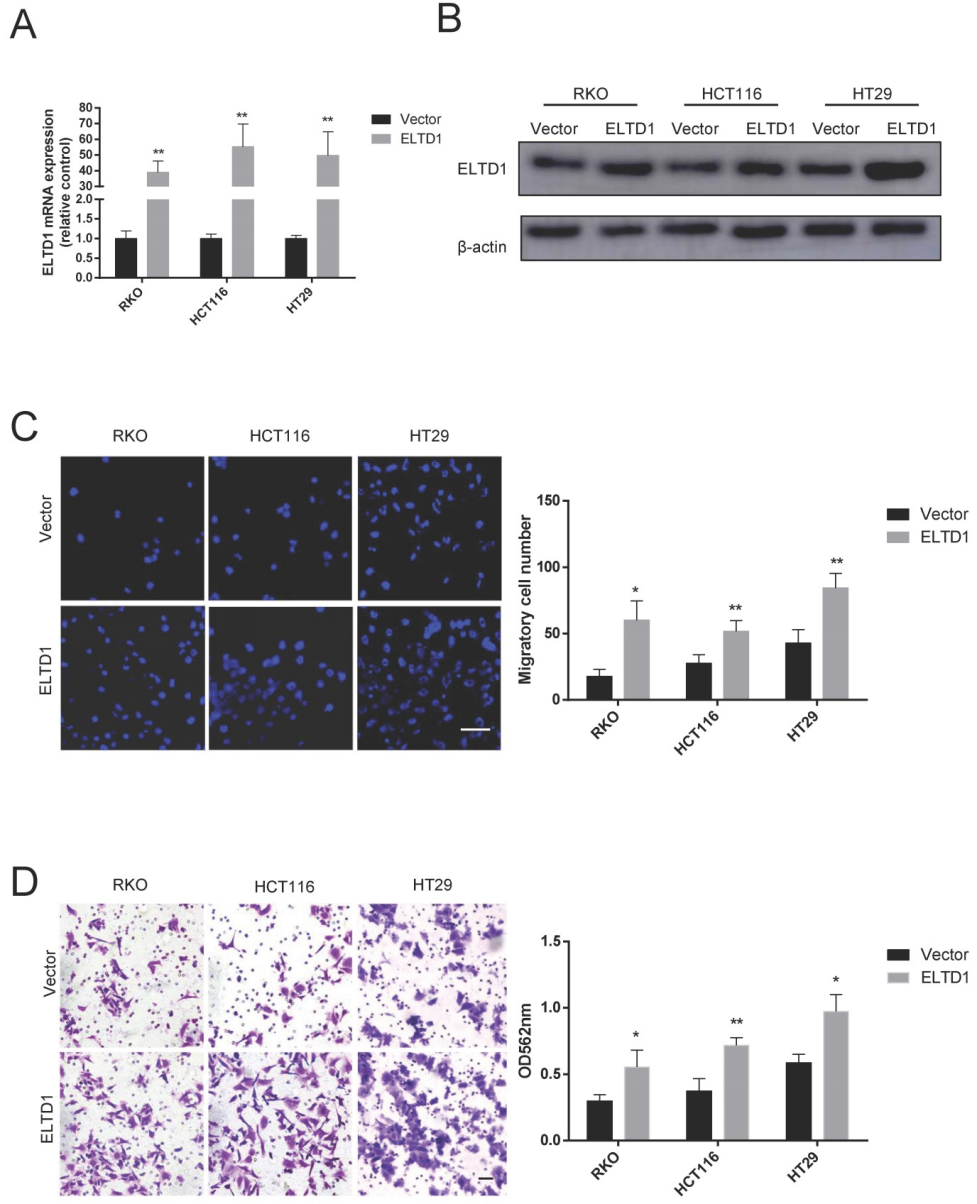
Overexpression of ELTD1 promoted CRC cell migration and invasion. **(A, B)** The mRNA and protein expression levels of ELTD1 were measured by qRT-PCR and Western blotting, respectively after transfection with the pEnCMV-ELTD1-3xFLAG plasmid. β-actin was used as the internal control.** (C)** Representative DAPI staining and statistical results of CRC cells in the Transwell assay.** (D)** Representative crystal violet staining and statistical results of CRC cells in the invasion assay. The results are shown as the mean ± standard deviation (SD) of three independent experiments. **p<0.01 and *p<0.05. Scale bar=100 μm.

To further determine the role of ELTD1 in the migration and invasion of CRC cells, HT-29, RKO and HCT116 cells were transfected with pEnCMV-ELTD1-3xFLAG to induce the expression of ELTD1 (Figure 4A, B). In addition, increased numbers of CRC cells migrated through the Transwell membrane (Figure 4C), and the numbers of CRC cells that invaded the Matrigel were also increased (Figure 4D). All these results indicated that ELTD1 might promote the migration and invasion of CRC cells.

### ELTD1 promoted the lung metastatic capacity of CRC cells in vivo

**Figure 5:**
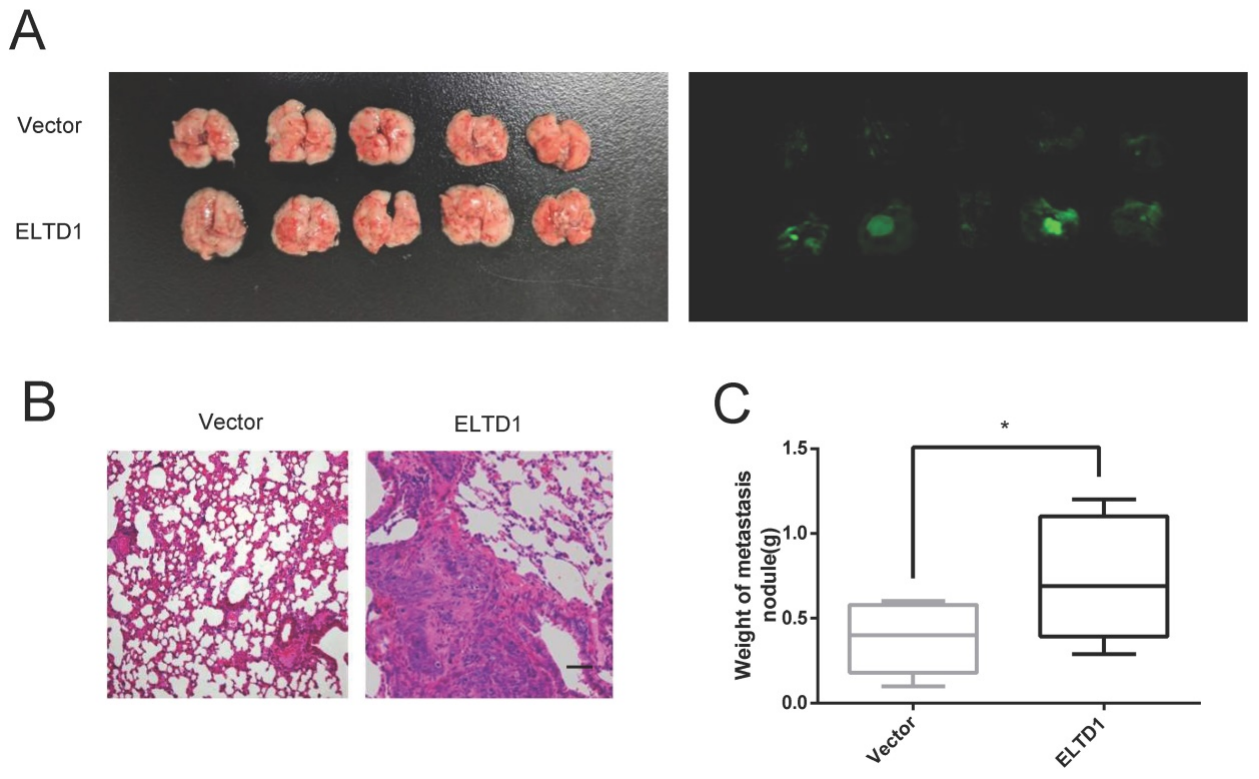
ELTD1 promoted CRC cell metastasis in vivo.** (A)** Representative gross photographs and in vivo fluorescence images of lung metastasis of mice treated with ELTD1 or vector as indicated. (n=5 for each group) **(B)** Representative HE-stained lung tissue samples. **(C)** Statistical results of metastasis nodule weight from the ELTD1 overexpression and control groups. Scale bar=100 μm.

To further confirm the association between ELTD1 expression and CRC metastasis in vivo, ELTD1-overexpressing HCT116 cells were transplanted into nude mice via tail vein injection. After 10 weeks, all the mice were sacrificed, and the lungs were harvested for H&E staining. The results showed that the nude mice treated with ELTD1-overexpressing HCT-116 cells exhibited more lung metastatic nodules than the corresponding control mice (Figure 5A-D). These data suggested that ELTD1 played a role in promoting CRC in vivo.

### ELTD1 regulated the expression of MMP2 at the transcriptional level

**Figure 6:**
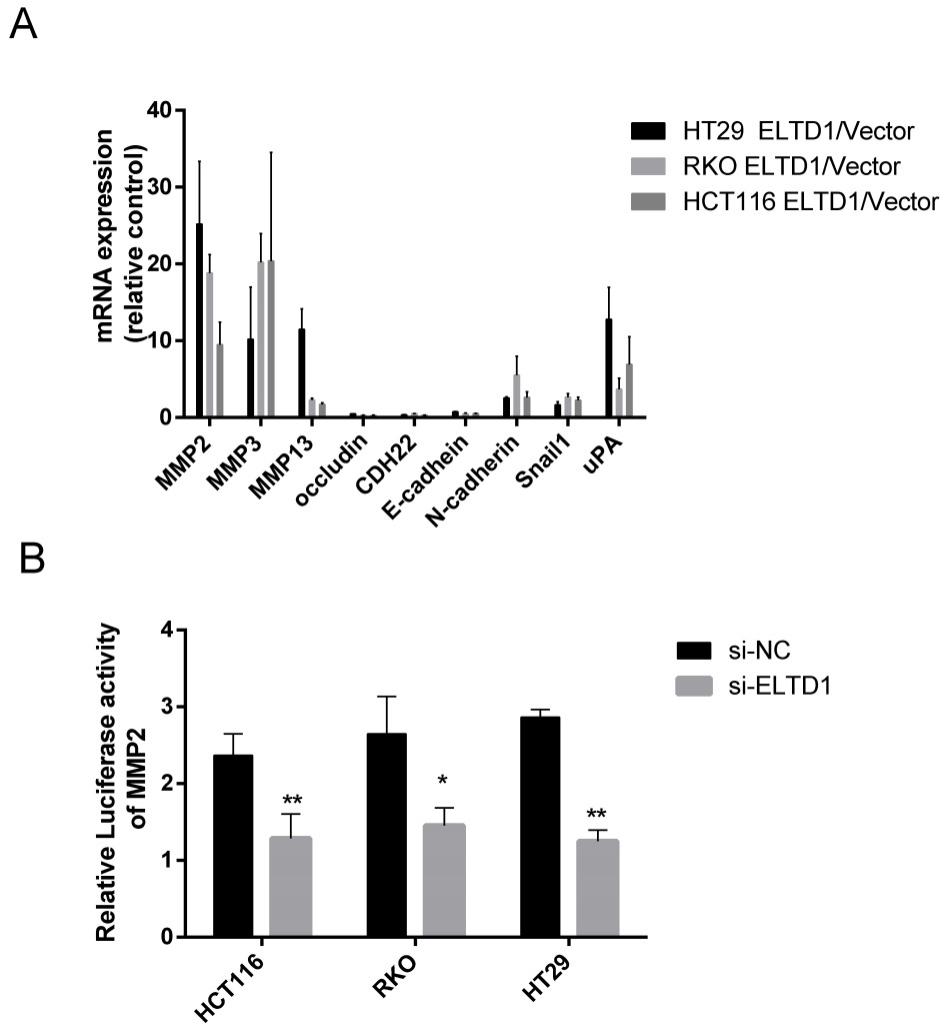
ELTD1 regulated the expression of MMP2 at the transcriptional level.** (A)** mRNA expression levels of invasiveness-related genes in CRC cells were measured after ELTD1 knockdown.** (B)** Transcriptional activity of the MMP2 promoter was assessed after silencing ELTD1 expression in HT29, RKO and HCT116 cells. The results are shown as the mean ± standard deviation (SD) of three independent experiments. **p<0.01, *p<0.05

To study how ELTD1 regulates cell migration and invasion, the expression of genes related to invasiveness was measured in both ELTD1-silenced and ELTD1-overexpressing CRC cells. Classic metastasis- and invasion-associated genes, including the epithelial cell biomarkers E‐cadherin and occluding and N-cadherin, matrix metalloproteinases MMP2, MMP3, and MMP9, zinc-finger transcription factor Snail1, and metastasis-related transmembrane glycoprotein CDH22, were assessed. Silencing ELTD1 in CRC cells downregulated MMP2, MMP3, MMP9, N-cadherin and Snail1 expression and upregulated E-cadherin, occludin and CDH22 expression. Among these molecules, the down-regulation of MMP2 expression was most remarkable (Figure 6A), and dramatic up-regulation of MMP2 expression was observed in ELTD1-overexpressing CRC cells (Supplementary Figure 3). To explore how ELTD1 regulates the expression of MMP2 in CRC cells, we detected luciferase activity using a dual-luciferase reporter assay. The results showed that the relative firefly/Renilla luciferase activity was much lower in CRC cells transfected with ELTD1 siRNA than in those transfected with negative control siRNA (Figure 6B).

### ELTD1 promoted migration and invasion via MMP2

**Figure 7:**
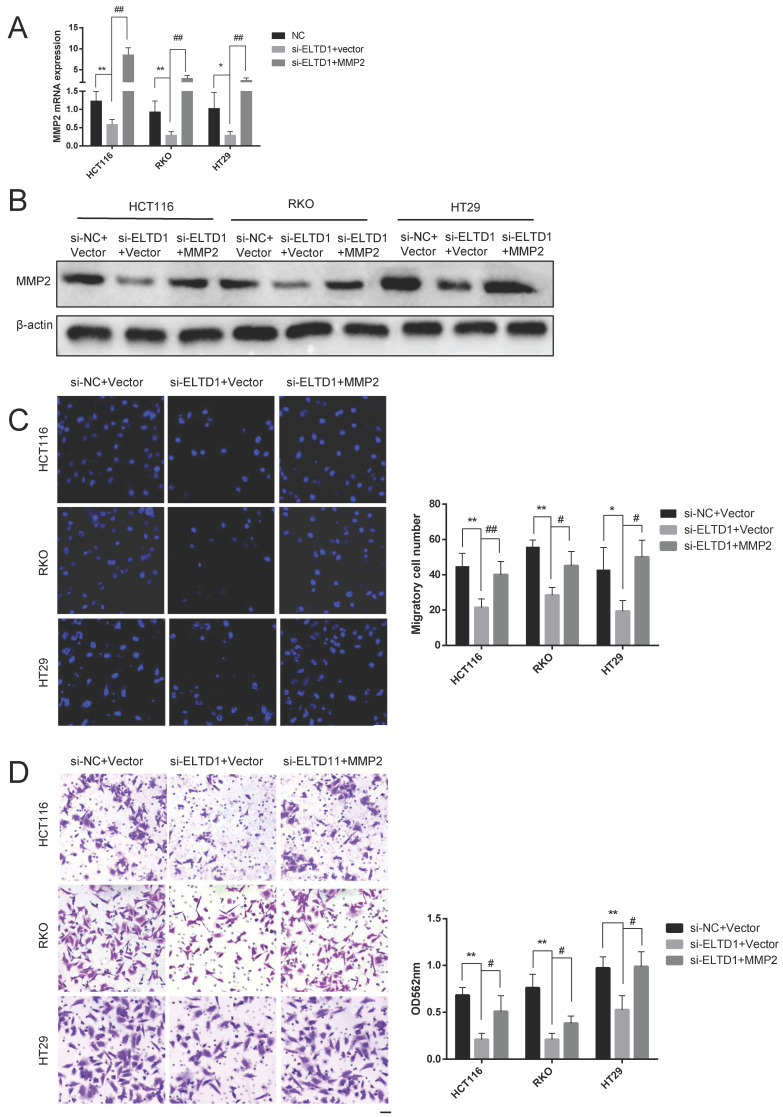
ELTD1 actively regulated the migration and invasion of CRC cells through MMP2. **(A, B)** The efficiency of the cotransfection of ELTD1 siRNA and MMP2-expressing plasmids into HT29, RKO and HCT116 cells was verified by qRT-PCR and Western blotting.** (C, D)** Transwell migration and invasion results of HT29 and RKO cells cotransfected with the MMP2 expression vector and si-ELTD1. The results are shown as the mean ± standard deviation (SD) of three independent experiments. **p<0.01, *p<0.05, ^##^p<0.01 and ^#^p<0.05. Scale bar=100 μm.

To elucidate whether the role of ELTD1 in the migration and invasion of CRC cells was dependent on MMP2, ELTD1 siRNA and an MMP2-expressing plasmid were cotransfected into CRC cells. qRT-PCR and Western blotting analyses consistently verified the transfection efficiency (Figure 7A and B). The Transwell migration assays showed that the decrease in the number of CRC cells migrating through the Transwell membrane after transfection with ELTD1 siRNA was reversed by transfection with the MMP2 plasmid (Figure 7C). In addition, the Transwell invasion assay yielded similar results (Figure 7D). All these results indicated that the role of ELTD1 in the migration and invasion of CRC cells was dependent on MMP2.

## Discussion

The studies of AOM+DSS mice and clinical samples CRC and the analysis of TCGA bioinformatics database in this study showed that ELTD1 expression was notably upregulated in CRC and correlated with lymph node metastasis and poor prognosis; these results suggest that ELTD1 can be used as a prognostic marker for CRC. Previous studies have reported that changes in the expression of several types of aGPCRs can be observed in various solid tumors. The expression of CD97/ADGRE5, a member of the aGPCR family, was significantly upregulated in breast, thyroid, stomach, pancreas, esophageal cancer and CRC and was associated with the metastasis of a variety of tumors 17-19. The expression of GPR56/ADGRL1, another member of the aGPCR family, was upregulated in CRC tissues 20, 21. Abdul Aziz et al 22 analyzed the gene expression profiles of tumor tissues from 78 CRC patients by microarray and followed the survival time of these patients. A 19-gene expression signature, including elevated ELTD1 expression, predicted a poor prognosis in CRC, and this signature was more accurate than the traditional Dukes staging. However, another study that included 111 CRC patients (12 with a staining score of 4 and 99 with a staining score of 3) showed that the prognosis was better with higher ELTD1 expression 10. In our study, CRC patients with relatively high expression of ELTD1 had poor prognosis, as evidenced by the data of a larger sample obtained from TCGA. The reason for the inconsistent conclusion may be due to different sample sources and sample sizes.

Members of the aGPCR family can regulate the migration and invasion abilities of tumor cells. Steinert et al. also found that CD97 promoted cell migration and invasion in 15 different CRC cell lines^23^. The role of ELTD1 in the migration and invasion of cancer cells has been reported in several previous studies. Li et al 24 found that ELTD1 promoted the migration and invasion of glioma cells. Another study showed that ELTD1 could promote the migration of liver cancer cells, which was impaired when the cells were cocultured with cancer-related fibroblasts (CAFs) 25, indicating that the effect of ELTD1 on liver cancer cells might be related to the tumor microenvironment. However, the effect of ELTD1 on the invasion and metastasis of CRC is still unclear. After evaluating the gain and loss of function of ELTD1 in CRC, we found that ELTD1 could promote the invasiveness of CRC cells both in vivo and in vitro.

To date, the corresponding ligand of ELTD1 has not been identified, making it an orphan receptor. In addition, the downstream signal transduction pathways and genes of ELTD1 in tumors are still not well known. In glioma, ELTD1 promotes cell migration and invasion by activating the JAK/STAT3/HIF-1α signaling axis 24. MMP2 can degrade most components of the extracellular matrix and basement membrane 26, 27 and plays an important role in regulating tumor invasion and metastasis 28-30. Our study showed that ELTD1 silencing in CRC cells significantly downregulated MMP2 expression, and overexpression of MMP2 partially reversed the inhibition of migration and invasion by ELTD1 siRNA, proving that MMP2 was an important downstream target of ELTD1.

Previously, several studies identified a series of upstream transcription factors that target MMP2. Through ChIP and luciferase promoter assays, Teng et al 31 found that HMGA1 can bind to the promoter region of MMP2 and promote the transcription of MMP2 in liver cancer cells. Xiang et al 32 found that the ERα-36-STAT3 complex could directly bind to the promoter regions of MMP2/9 and promote their expression in breast cancer cells. In another study, ChIP analyses indicated that MMP2 transcription was directly regulated by RARα 33. We revealed in this study that ELTD1 could activate the luciferase activity of MMP2 in CRC cells. As ELTD1 is not a transcription factor, the transcriptional regulation of MMP2 by ELTD1 might be achieved in an indirect manner. To verify the transcription factors involved in the regulation of MMP2 transcription by ELTD1, DNA pull-down with the MMP2 promoter followed by protein profiling analysis will be performed in future studies.

Taken together, the results of this study showed that ELTD1 could affect the migration and invasion of CRC cells by targeting MMP2. ELTD1 was a potential therapeutic target of CRC.

## Supplementary Material

Supplementary figures.

## Figures and Tables

**Table 1:** Relationship between ELTD1 expression and clinicopathologic features of CRC patients (n = 86)

Features	ELDT1 expression		χ2	P Value
	Low(n = 52)	High(n = 34)		
Age				
≤60	16	12	0.192	0.662
>60	36	22		
Gender				
Male	23	18	0.625	0.429
Female	29	16		
Invasion depth				
T1+T2	3	1	0.371	0.543
T3+T4	49	33		
Lymph metastasis				
N0	27	10	4.25	0.039*
N1+N2	25	24		
Distant metastasis				
M0	37	20	2.521	0.112
M1	15	14		
TNM stage				
I-II	30	16	0.934	0.334
III-IV	22	18		

*P < 0.05.
