# Supplementary material for: ELTD1 promotes invasion and metastasis by activating MMP2 in colorectal cancer
Source: Int J Biol Sci. 2021 Jul 13;17(12):3048–58. doi: 10.7150/ijbs.62293 (PMC8375227; doi:10.7150/ijbs.62293)
Supplement: Supplementary file 1 — Supplementary figures. [file ijbsv17p3048s1.pdf]

Supplementary Figure 1

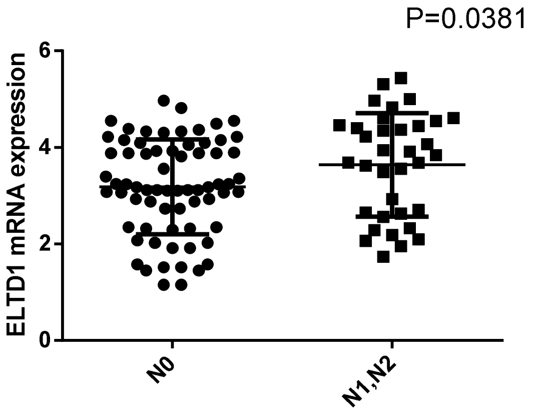

Supplementary Figure 2

A

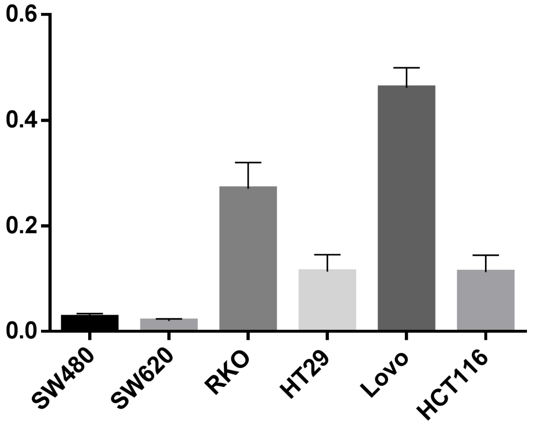

B

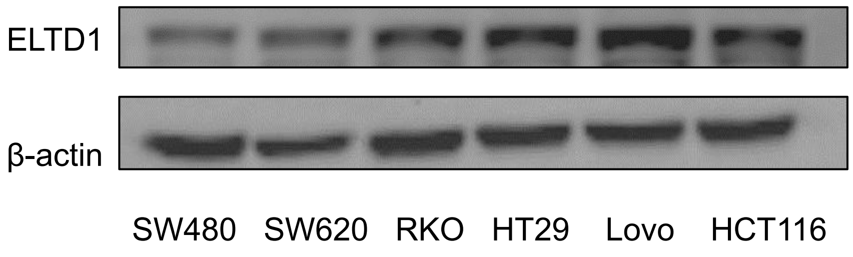

Supplementary Figure 3

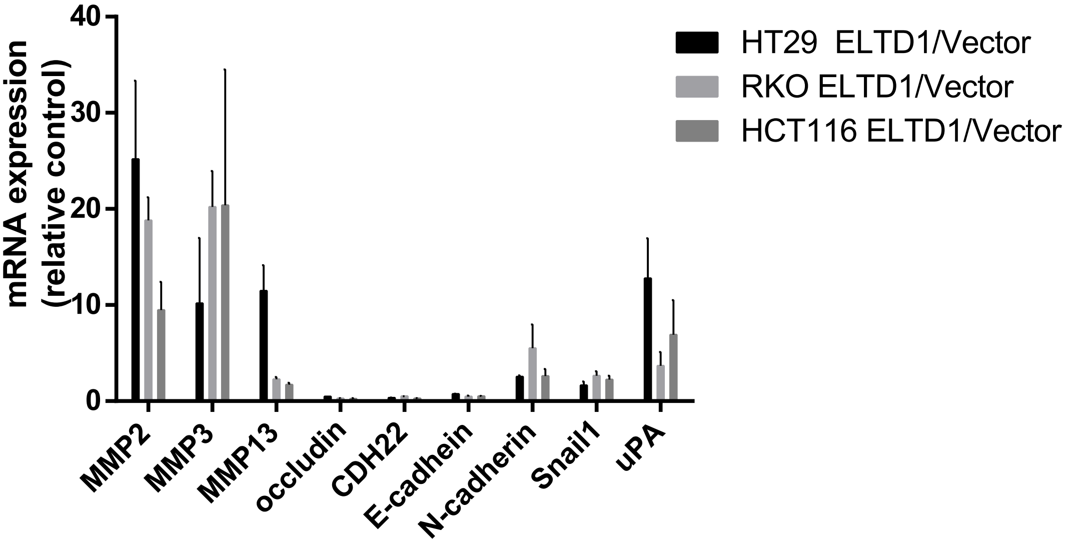

Supplementary Figure 1 mRNA expression of ELTD1 in CRC tissues with different N stages. (n=77)

Supplementary Figure 2 (A, B) The mRNA and protein expression levels of ELTD1 in wild-type CRC cells were measured by qRT-PCR and Western blotting. The results are shown as the mean ± standard deviation (SD) of three independent experiments.

Supplementary Figure 3 mRNA expression levels of invasiveness-related genes in CRC cells were measured after ELTD1 overexpression. The results are shown as the mean ± standard deviation (SD) of three independent experiments.
